# Preventing P-gp Ubiquitination Lowers Aβ Brain Levels in an Alzheimer’s Disease Mouse Model

**DOI:** 10.3389/fnagi.2018.00186

**Published:** 2018-06-26

**Authors:** Anika M. S. Hartz, Yu Zhong, Andrew N. Shen, Erin L. Abner, Björn Bauer

**Affiliations:** ^1^Sanders-Brown Center on Aging, University of Kentucky, Lexington, KY, United States; ^2^Department of Pharmacology and Nutritional Sciences, University of Kentucky, Lexington, KY, United States; ^3^Department of Pharmaceutical Sciences, College of Pharmacy, University of Kentucky, Lexington, KY, United States

**Keywords:** blood-brain barrier, P-glycoprotein, Alzheimer’s disease, brain capillaries, amyloid beta, ubiquitin-proteasome system, ubiquitin, proteasome

## Abstract

One characteristic of Alzheimer’s disease (AD) is excessive accumulation of amyloid-β (Aβ) in the brain. Aβ brain accumulation is, in part, due to a reduction in Aβ clearance from the brain across the blood-brain barrier. One key element that contributes to Aβ brain clearance is P-glycoprotein (P-gp) that transports Aβ from brain to blood. In AD, P-gp protein expression and transport activity levels are significantly reduced, which impairs Aβ brain clearance. The mechanism responsible for reduced P-gp expression and activity levels is poorly understood. We recently demonstrated that Aβ_40_ triggers P-gp degradation through the ubiquitin-proteasome pathway. Consistent with these data, we show here that ubiquitinated P-gp levels in brain capillaries isolated from brain samples of AD patients are increased compared to capillaries isolated from brain tissue of cognitive normal individuals. We extended this line of research to *in vivo* studies using transgenic human amyloid precursor protein (hAPP)-overexpressing mice (Tg2576) that were treated with PYR41, a cell-permeable, irreversible inhibitor of the ubiquitin-activating enzyme E1. Our data show that inhibiting P-gp ubiquitination protects the transporter from degradation, and immunoprecipitation experiments confirmed that PYR41 prevented P-gp ubiquitination. We further found that PYR41 treatment prevented reduction of P-gp protein expression and transport activity levels and substantially lowered Aβ brain levels in hAPP mice. Together, our findings provide *in vivo* proof that the ubiquitin-proteasome system mediates reduction of blood-brain barrier P-gp in AD and that inhibiting P-gp ubiquitination prevents P-gp degradation and lowers Aβ brain levels. Thus, targeting the ubiquitin-proteasome system may provide a novel therapeutic approach to protect blood-brain barrier P-gp from degradation in AD and other Aβ-based pathologies.

## Introduction

Accumulation of amyloid-β (Aβ) in the brain is a neuropathological hallmark of Alzheimer’s disease (AD; Hardy and Selkoe, 2002). Increasing evidence suggests that Aβ brain accumulation is due to impaired Aβ clearance from the brain (Zlokovic, 2005; Mawuenyega et al., 2010; Wang et al., 2016). Several studies indicate a role for the blood-brain barrier efflux transporter P-glycoprotein (P-gp) in clearing Aβ from brain to blood: (1) P-gp transports Aβ *in vitro* (Lam et al., 2001; Kuhnke et al., 2007; Hartz et al., 2010); (2) in mice lacking P-gp, Aβ clearance is decreased while Aβ brain levels are increased (Cirrito et al., 2005; Yuede et al., 2016); (3) P-gp expression and transport activity are reduced at the blood-brain barrier of several AD mouse models (Hartz et al., 2010; Mehta et al., 2013; Park et al., 2014); and (4) treating human amyloid precursor protein (hAPP) mice with the PXR activator pregnenolone-16-carbonitrile (PCN) induces P-gp expression and activity, which restores Aβ transport and reduces Aβ brain levels (Hartz et al., 2010). (5) Results of multiple studies from different laboratories show that P-gp protein expression levels at the human blood-brain barrier are significantly reduced in AD patients (Wijesuriya et al., 2010; Jeynes and Provias, 2011; Carrano et al., 2014; Chiu et al., 2015). Consistent with reduced P-gp protein expression levels, results of recent PET imaging studies indicate compromised P-gp transport activity in AD patients compared to age-matched cognitive healthy individuals (van Assema et al., 2012; Deo et al., 2014). Thus, existing studies support the conclusion that blood-brain barrier P-gp is reduced in AD, however, more insights into the mechanism that triggers this phenomenon are needed to prevent P-gp loss in AD and improve Aβ brain clearance.

In this regard, we recently reported that exposing isolated rat brain capillaries to Aβ_40_ at concentrations similar to those found in AD patients reduced P-gp protein expression and transport activity levels in a time- and concentration-dependent manner (Hartz et al., 2016). We showed that Aβ_40_ triggers ubiquitination, internalization, and proteasomal degradation of P-gp in isolated rat brain capillaries *ex vivo* (Akkaya et al., 2015; Hartz et al., 2016). Collectively, these results indicate that blood-brain barrier P-gp is part of an Aβ clearance system and that P-gp expression and transport activity levels are reduced in AD, suggesting a link between high Aβ levels and reduced brain capillary P-gp levels in AD pathology.

In the present study, we show that P-gp protein expression and transport activity levels are reduced and that P-gp protein is highly ubiquitinated in isolated human brain capillaries from AD patients compared to P-gp in brain capillaries isolated from age-matched cognitive normal individuals. We extended our previous *ex vivo* findings to *in vivo* studies with hAPP mice by blocking P-gp ubiquitination in hAPP mice, which is the initial step of protein degradation mediated by the ubiquitin-proteasome system. We used transgenic hAPP-overexpressing mice (Tg2576) to test the hypothesis that preventing P-gp reduction results in a reduction of Aβ brain levels. We show here that inhibiting P-gp ubiquitination *in vivo* with PYR41, a cell-permeable, irreversible inhibitor of the ubiquitin-activating enzyme E1, prevents P-gp reduction at the blood-brain barrier and significantly lowers Aβ brain levels *in vivo*.

Together, our findings suggest that targeting the ubiquitin-proteasome system by inhibiting ubiquitination protects brain capillary P-gp and thereby lowers Aβ brain levels.

## Materials and Methods

### Experimental Design and Statistical Analysis

Sample size (animal numbers, number of brain capillaries, number of human tissue samples) for individual experiments were based on power analyses of preliminary data and previously published data (Hartz et al., 2010, 2016, 2017), and are given in the corresponding figure legends. Number of repetitions are stated in the results section and the figure legends.

Results are presented as mean ± SEM. One-way analysis of variance (ANOVA) was used to assess differences in group means. Pre-planned pairwise *post hoc* tests were carried out when the overall F test was significant, and the Bonferroni correction was used to control the type 1 error rate. Statistical significance was set at α = 0.05. Data were analyzed using GraphPad Prism^®^ statistical software (version 7.00; RRID:SCR_002798).

### Animals

All animal experiments were approved by the University of Kentucky Institutional Animal Care and Use Committee (Protocol #2014-1233; PI: AMS Hartz) and carried out in accordance with AAALAC regulations, the US Department of Agriculture Animal Welfare Act, and the Guide for the Care and Use of Laboratory Animals of the NIH.

Male transgenic hAPP-overexpressing mice (Tg2576 strain; 129S6.Cg-Tg(APPSWE)2576Kha; RRID:IMSR_TAC:2789; *n* = 45) and corresponding male wild type (WT) mice (*n* = 15; RRID:IMSR_TAC:2789) were purchased from Taconic Farms (Germantown, NY, USA). On arrival, mice were 8-week old with an average body weight of 27.1 ± 2.6 *g* (SD) for WT mice and 29.9 ± 2.9 *g* (SD) for hAPP mice. Animals were single-housed in an AALAC-accredited temperature- and humidity-controlled vivarium (23°C, 60%–65% relative humidity, 14:10 light-dark cycle) in cages connected to an EcoFlo Allentown ventilation system (Allentown Inc., Allentown, NJ, USA). Animals had *ad libitum* access to tap water and standard rodent feed (Harlan Teklad Chow 2918, Harlan Laboratories Inc., Indianapolis, IN, USA) and were allowed to habituate to the vivarium for at least 2 weeks after arrival before the start of experiments.

### Human Brain Tissue Samples

Human brain tissue samples (inferior parietal lobule) were obtained from the UK-ADC tissue bank (IRB #B15-2602-M). Case inclusion criteria for this study were enrollment in the UK-ADC longitudinal autopsy cohort (Nelson et al., 2007), a post-mortem interval ≤4 h, and a final consensus diagnosis determined by a group of UK-ADC neuropathologists, neuropsychologists, and neurologists. Cases were classified into two groups: Group (1) cognitive normal (*n* = 3; a classification of “normal” denotes a consensus diagnosis of normal cognition and CERAD rating of “criteria not met”) and Group (2) AD patients (*n* = 3; Mirra et al., 1991). All brain tissue samples were from female individuals, whose average age at death was 85.5 ± 9.2 years (group 1, cognitive normal, post-mortem interval: 1.5 h ± 0.3 h, Braak stage score: 1 ± 0) and 86.5 ± 9.2 years (group 2, AD, post-mortem interval: 3.5 h ± 0.7 h, Braak stage score: 5.5 ± 0.7).

### Chemicals

Antibodies against β-actin (ab8226; RRID:AB_306371), human Aβ40 (ab12265; RRID:AB_298985), human Aβ42 (ab12267; RRID:AB_298987), LRP (ab92544; RRID:AB_2234877), RAGE (ab3611; RRID:AB_303947), APP (ab11118; RRID:AB_442855) and 20S proteasome (ab109530; RRID:AB_10860339; antibody raised against a synthetic peptide within the human proteasome 20S C2 unit (amino acids: 250–350; C terminal)), as well as cyclosporine A (CSA; ab120114) were purchased from Abcam (Cambridge, MA, USA). Modified Dulbecco’s phosphate buffered saline (DPBS; with 0.9 mM Ca^2+^ and 0.5 mM Mg^2+^) was purchased from HyClone (Logan, UT, USA). Complete™ protease inhibitor was purchased from Roche (Mannheim, Germany). C219 antibody against P-gp was purchased from ThermoFisher (MA126528; RRID:AB_795165; Waltham, MA, USA). Fluorescein-hAβ_42_ [fluorescein-Aβ_(1–42)_] was purchased from rPeptide (Bogart, GA, USA). [N- (4-nitrobenzofurazan-7-yl)-D-Lys8]-cyclosporine A (NBD-CSA) was custom-synthesized by R. Wenger (Basel, Switzerland; Wenger, 1986). PSC833 was a kind gift from Novartis (Basel, Switzerland). PYR41, CelLytic™ M, Ficoll^®^ PM 400, bovine serum albumin and all other chemicals were purchased at the highest grade from Sigma-Aldrich (St. Louis, MO, USA).

### PYR41 Dosing

Table 1 shows the dosing regimen for this *in vivo* study. Mice were dosed as follows: Group 1: WT mice (*n* = 15) received *i.p*. vehicle every third day and *p.o*. vehicle on both days between *i.p*. vehicle injections. Group 2: hAPP mice (*n* = 15) also received *i.p*. vehicle every third day and *p.o*. vehicle on both days between *i.p*. vehicle injections. Group 3: hAPP-PYR41 mice (*n* = 15) were dosed every third day with 2 mg/kg PYR41 by *i.p*. injection and received *p.o*. vehicle on days between PYR41 treatment. Group 4: hAPP-PYR41/CSA mice (*n* = 15) were dosed every third day with 2 mg/kg PYR41 by *i.p*. injection and received 25 mg/kg CSA via oral gavage on both days between doses of PYR41.

**Table 1:** PYR41 dosing regimen.

Group	Day of treatment													
	1	2	3	4	5	6	7	8	9	10	11	12	13	14
**WT**	Vehicle	Vehicle	Vehicle	Vehicle	Vehicle	Vehicle	Vehicle	Vehicle	Vehicle	Vehicle	Vehicle	Vehicle	Vehicle	Vehicle
	(ip)	(po)	(po)	(ip)	(po)	(po)	(ip)	(po)	(po)	(ip)	(po)	(po)	(ip)	(po)
**hAPP**	Vehicle	Vehicle	Vehicle	Vehicle	Vehicle	Vehicle	Vehicle	Vehicle	Vehicle	Vehicle	Vehicle	Vehicle	Vehicle	Vehicle
	(ip)	(po)	(po)	(ip)	(po)	(po)	(ip)	(po)	(po)	(ip)	(po)	(po)	(ip)	(po)
**hAPP-**	**PYR41**	Vehicle	Vehicle	**PYR41**	Vehicle	Vehicle	**PYR41**	Vehicle	Vehicle	**PYR41**	Vehicle	Vehicle	**PYR41**	Vehicle
**PYR41**	**(ip)**	(po)	(po)	**(ip)**	(po)	(po)	**(ip)**	(po)	(po)	**(ip)**	(po)	(po)	**(ip)**	(po)
**hAPP-**	**PYR41**	CSA	CSA	**PYR41**	CSA	CSA	**PYR41**	CSA	CSA	**PYR41**	CSA	CSA	**PYR41**	CSA
**PYR41/**	**(ip)**	(po)	(po)	**(ip)**	(po)	(po)	**(ip)**	(po)	(po)	**(ip)**	(po)	(po)	**(ip)**	(po)
**CSA**

*The present dosing regimen was based on a 2-week preliminary dosing study with CD-1 mice (data not shown). WT and hAPP mice were treated according to the following dosing scheme over the course of 14 days: WT and hAPP mice were given vehicle p.o. by oral gavage or by i.p. injection once every 3 days. hAPP-PYR41 mice received 2 mg/kg PYR41 by i.p. injection once every 3 days and vehicle p.o. by oral gavage on all other days. hAPP-PYR41/CSA mice received 2 mg/kg PYR41 by i.p. injection once every 3 days and 25 mg/kg CSA p.o. by oral gavage on all other days*.

### Blood Collection

Blood samples were collected by facial vein bleeding 24 h prior to the start of the first dose to obtain control values. Twenty-four hours after the last dose, mice were euthanized by CO_2_ inhalation, decapitated and trunk blood was collected in heparinized blood collection tubes. Plasma was obtained by centrifugation at 5000× *g* for 15 min at 4°C and stored at −80°C until further analysis.

### Brain Capillary Isolation

Brain capillaries were isolated using a modified method previously described elsewhere (Hartz et al., 2012). Briefly, mice were euthanized with CO_2_ followed by decapitation. Brains were removed, cleaned, dissected and homogenized in DPBS containing Ca^2+^/Mg^2+^ and supplemented with 5 mM D-glucose and 1 mM sodium pyruvate. Ficoll^®^ PM 400 was added to the homogenized brains (final concentration 15%) and the homogenate was centrifuged (5800 *g*, 20 min, 4°C). After centrifugation, the capillary-enriched pellet was collected and resuspended in 1% BSA-DPBS. The capillary suspension was first passed through a 300 μm nylon mesh and then passed over a glass bead column using 1% BSA-DPBS. Capillaries adhering to the glass beads were washed off and collected by agitation in 1% BSA-DPBS. After centrifugation (1500 *g*, 3 min, 4°C), the capillary pellet was washed three times with DPBS (no BSA), collected, and used for experiments or crude membrane isolation.

### Brain Capillary Crude Membrane Isolation

Brain capillary crude membranes were isolated as previously described (Hartz et al., 2012). Freshly isolated brain capillaries were homogenized in lysis buffer (CelLytic™ M, Sigma-Aldrich, St. Louis, MO, USA) containing Complete™ protease inhibitor. Homogenates were centrifuged to separate the membrane fraction from organelles and debris (10,000 *g*, 15 min, 4°C), and the resulting membrane-containing supernatant was centrifuged to pellet capillary crude membranes (100,000 *g*, 90 min, 4°C). The resulting pellet containing brain capillary crude membranes was resuspended and stored at −80°C.

### Aβ Immunostaining of Brain Capillaries

Aβ immunostaining of mouse brain capillaries was performed as previously described (Hartz et al., 2010). Briefly, isolated mouse brain capillaries were fixed with 3% paraformaldehyde/0.25% glutaraldehyde for 30 min at room temperature. After washing with PBS, capillaries were permeabilized with 0.5% Triton X-100 for 30 min and washed with PBS. Capillaries were blocked with 1% BSA/DPBS for 60 min and incubated overnight at 4°C with a 1:250 (4 μg/ml) dilution of rabbit polyclonal antibody to human Aβ1–40 (hAβ40; ab12265, Abcam, Cambridge, MA, USA; RRID:AB_298985) or rabbit polyclonal to human Aβ1–42 antibody (hAβ42; ab12267; Abcam, Cambridge, MA, USA; RRID:AB_298987). Capillaries were washed with 1% BSA/PBS and incubated with Alexa-Fluor 488-conjugated goat anti-rabbit IgG (1:1000, 1 μg/ml; Invitrogen, Carlsbad, CA, USA; RRID:AB_2576217) for 1 h at 37°C. Nuclei were counterstained with 1 μg/ml 4,6-diamidino-2-phenylindole (DAPI; MilliporeSigma, Burlington, MA, USA; RRID:SCR_014366). Aβ immunofluorescence was visualized with confocal microscopy (Leica TCS SP5 confocal microscope, 63× water objective, NA 1.2, Leica Instruments, Wetzlar, Germany).

From each treatment group, confocal images of seven capillaries were acquired. Aβ membrane immunofluorescence for each capillary was quantitated with ImageJ software v1.48 as previously described (Hartz et al., 2010). A 10 × 10 grid was superimposed on each image, and fluorescence measurements of capillary membranes were taken between intersecting grid lines. Fluorescence intensity for each capillary was the mean of three measurements per capillary.

### Aβ ELISA

Human Aβ40 and Aβ42 levels were quantitated in plasma and brain samples by ELISA (KHB3482 (Sensitivity: <6 pg/ml) and KHB3442 (Sensitivity: <10 pg/ml) from Invitrogen, Camarillo, CA, USA) according to the manufacturer’s protocol.

### Plasma Samples

Blood samples were collected from control, PYR41 and PYR41/CSA-treated hAPP transgenic mice. Plasma was obtained from blood samples by centrifugation at 5000 *g* for 5 min at 4°C, and then diluted with standard diluent buffer provided with the ELISA kit. To determine hAβ40 levels, samples were diluted 1:50; to determine hAβ42 levels, samples were diluted 1:4.

### Brain Samples

To determine brain hAβ40 and hAβ42 levels, brain tissue samples were homogenized in guanidine Tris-HCl buffer (5 M, pH 8) to extract Aβ. To determine hAβ40 levels, samples were diluted 1:20 in DPBS buffer containing 5% BSA and 0.03% Tween-20; to determine hAβ42 levels, samples were diluted 1:5. Diluted samples were centrifuged at 16,000 *g* for 20 min at 4°C; the supernatant was used for ELISA analysis.

Absorbance was measured at 450 nm using a Synergy™ H1 Hybrid Multi-Mode Reader (BioTek, Winooski, VT, USA). A standard curve was plotted using Gen5™ software v2.07 to determine the concentration of hAβ40 and hAβ42 in plasma and brain samples; values at 450 nm were corrected for background absorbance; four parameter logistic ELISA curve fitting was selected.

### Western Blotting

Protein expression levels from various tissues were determined by Western blotting as described previously (Hartz et al., 2010, 2016). Protein concentration of brain capillary crude membranes was measured with the Bradford assay. Western blots were performed by using the Invitrogen NuPage^®^ Bis-Tris electrophoresis and blotting system. After protein electrophoresis and transfer, blotting membranes were blocked and incubated overnight with the primary antibody as indicated. Membranes were washed and incubated for 1 h with horseradish peroxidase-conjugated ImmunoPure secondary IgG antibody (1:5000, 0.15 μg/ml; Thermo Fisher Scientific, Waltham, MA, USA). Proteins were detected with SuperSignal West Pico Chemoluminescent Substrate (Thermo Fisher Scientific, Waltham, MA, USA). Protein bands were visualized and recorded with a Bio-Rad ChemiDoc XRS+ gel documentation system (Bio-Rad Laboratories, Hercules, CA, USA). Image Lab 5.0 software from Bio-Rad Laboratories (RRID:SCR_014210) was used for densitometric analyses of band intensities and digital molecular weight analyses; the molecular weight marker was RPN800E (GE Healthcare, Chalfont St. Giles, Buckinghamshire, UK). Linear adjustments of contrast and brightness were applied to entire Western blot images. None of the Western blots shown were modified by nonlinear adjustments.

### Immunoprecipitation

Immunoprecipitation was carried out as previously reported (Hartz et al., 2016). Briefly, brain capillaries were homogenized in lysis buffer (CelLytic™ M, Sigma-Aldrich, St. Louis, MO, USA) containing Complete™ protease inhibitor. Samples were centrifuged to separate cellular membranes from organelles and debris (10,000 *g*, 15 min, and 4°C). Protein concentration of the supernatants was determined by Bradford assay.

Anti-P-gp antibody (C219, EMD Millipore, Billerica, MA, USA) was coupled with Pierce Protein A/G Plus Agarose (Thermo Fisher Scientific, Waltham, MA, USA) beads overnight at 4°C in TBST (20 mM Tris (pH 8.0), 170 mM NaCl, 0.05% Tween 20) supplemented with 1% BSA. The immune complex was added to the cell lysate and incubated for 3 h at 4°C. Beads were washed with lysis buffer (10 mM Tris (pH 7.5), 2 mM EDTA, 100 mM NaCl, 1% NP-40, 50 mM NaF, 1 mM Na_3_VO_4_) containing Complete™ protease inhibitor. For ubiquitin immunoprecipitations, the Pierce™ Ubiquitin Enrichment Kit (#89899; Thermo Fisher Scientific, Waltham, MA, USA) was used according to the manufacturer’s protocol. Immunoprecipitated proteins were eluted from agarose beads (IP: P-gp) or the ubiquitin affinity resin (IP: ubiquitin) with NuPAGE LDS sample buffer and heated at 70°C for 10 min. IP samples were resolved by SDS-PAGE and analyzed by Western blotting as described above.

### Simple Western Assay

Human brain capillary membrane samples were mixed with Wes™ sample buffer and analyzed with a Simple Western assay designed for the Wes™ instrument by ProteinSimple as previously described (San Jose, CA, USA; Hartz et al., 2016). All steps of the Wes™ Master Kit assay were performed according to the manufacturer’s protocol. Briefly, glass microcapillaries were loaded with stacking and separation matrices followed by sample loading. During capillary electrophoresis, proteins were separated by size and then immobilized to the capillary wall. P-gp and β-actin were identified with primary antibodies against P-gp (1:100, 3 μg/ml, C219, MA126528, ThermoFisher, Waltham, MA, USA; RRID:AB_795165) and β-actin (1:150, 5 μg/ml, ab8226, Abcam, Cambridge, MA, USA; RRID:AB_306371), respectively, followed by immunodetection using Wes™ Master Kit HRP conjugated anti-mouse secondary antibody and chemiluminescent substrate. Using Compass V. 2.6.5 software, electropherograms were generated for each sample and each protein (P-gp and β-actin). The area under the curve (AUC), which represents the signal intensity of the chemiluminescent reaction was analyzed for P-gp and β-actin. Values given for P-gp protein expression were normalized to β-actin.

### P-gp Transport Assay

To determine P-gp transport activity, freshly isolated brain capillaries from WT and hAPP mice were incubated for 1 h at room temperature with the fluorescent P-gp-specific substrate NBD-CSA (2 μM in PBS buffer; Hartz et al., 2004, 2008, 2010). To assess P-gp-mediated Aβ transport, isolated brain capillaries were incubated for 1 h at room temperature with 5 μM fluorescein-hAβ42 in DPBS buffer (Hartz et al., 2010). For each treatment, images of 10 capillaries were acquired by confocal microscopy using the 488 nm line of an argon laser (Leica Instruments, Wetzlar, Germany) of a Leica TCS SP5 confocal microscope with a 63× 1.2 NA water immersion objective. Images were analyzed by quantitating NBD-CSA fluorescence in the capillary lumen using Image J v.1.48v (Wayne Rasband, NIH, USA; RRID:SCR_003070). Specific, luminal NBD-CSA fluorescence was taken as the difference between total luminal fluorescence and fluorescence in the presence of the P-gp-specific inhibitor PSC833 (5 μM; Hartz et al., 2004, 2008, 2010).

## Results

### P-gp Ubiquitination Levels Are Increased in Brain Capillaries From AD Patients

**Figure 1:**
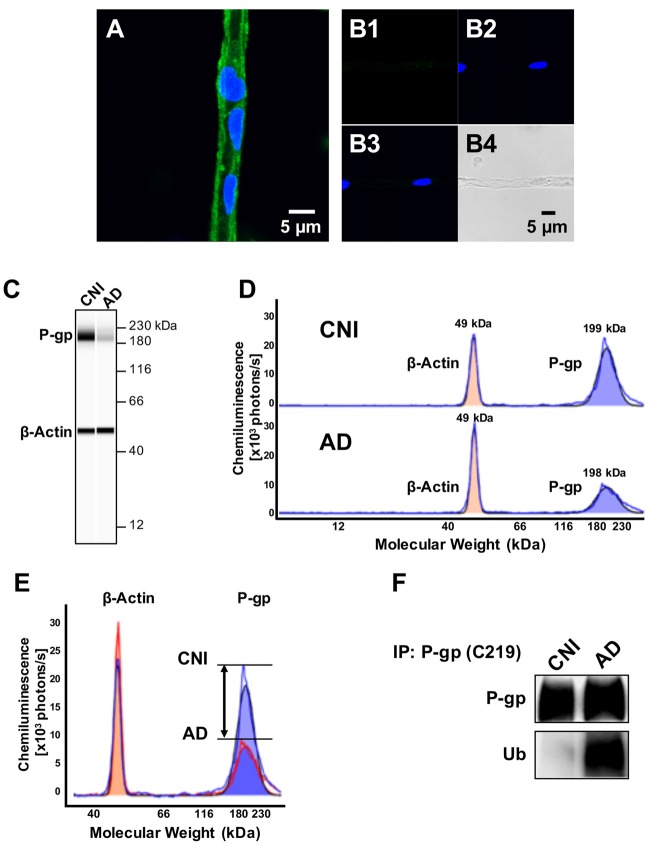
P-glycoprotein (P-gp) protein expression levels are decreased and P-gp ubiquitination levels are increased in brain capillaries from Alzheimer’s disease (AD) patients. **(A)** Representative image of a P-gp-immunostained (green) brain capillary isolated from brain tissue of a cognitive normal individual (CNI); nuclei were counterstained with DAPI (blue). **(B)** Negative control (no primary antibody): **(B1**; green channel); **(B2**; blue channel); **(B3**; overlay of green and blue channel) and **(B4**; transmitted light channel). **(C)** Representative WES™ image and **(D)** electropherogram showing reduced P-gp (blue shaded area) protein expression levels in brain capillaries isolated from human brain tissue (frontal cortex) of AD patients (*n* = 3) vs. CNIs (*n* = 3). **(E)** Overlay of electropherograms displayed in **(D)** show a reduction in the area under the curve (AUC) that represents P-gp protein expression levels (blue shaded area) in brain capillaries from AD patients (red line) relative to CNI (blue line). In contrast, β-actin levels (orange shaded area) in brain capillaries from AD patients (red line) and CNI (blue line) were the same. **(F)** Western blot showing that ubiquitin levels in P-gp-immunoprecipitates are increased in capillaries from AD patients compared to those from CNI.

Several studies have provided evidence showing that P-gp expression levels at the blood-brain barrier are reduced in AD patients compared to control individuals (Wijesuriya et al., 2010; Jeynes and Provias, 2011; Carrano et al., 2014; Chiu et al., 2015). To assess P-gp protein expression in human brain capillaries, we utilized a recently established protocol to isolate brain capillaries from fresh human frontal cortex tissue (Hartz et al., 2017). Figure 1 shows representative confocal images of a brain capillary isolated from brain tissue of a cognitive-normal individual (CNI) immunostained for P-gp (Figure 1A). The negative control (no primary antibody) shows no signal for P-gp (Figures 1B,B1: green channel; B2: blue channel; B3: overlay of green and blue channel; B4: transmitted light channel).

We utilized the Simple Western™ assay to quantitate P-gp protein levels in isolated human brain capillaries. This novel assay allows protein quantitation at 10-fold higher sensitivity and better reproducibility compared to Western blotting (Hartz et al., 2017). The assay is based on automated microcapillary electrophoresis; data robustness was tested at the NIH (Chen et al., 2015). Figure 1C shows that a band for P-gp was detected in membrane samples of brain capillaries isolated from CNI patients that was between the 180 kDa and 230 kDa bands of the molecular weight marker. Consistent with previous studies (Wijesuriya et al., 2010; Jeynes and Provias, 2011; Carrano et al., 2014; Chiu et al., 2015), we found that P-gp protein levels in brain capillary samples from AD patients were significantly lower compared to brain capillaries from cognitive normal individuals (CNI; Figure 1C); β-actin served as loading control. Figure 1D shows the electropherogram of the P-gp microcapillary electrophoresis. Analysis of this electropherogram revealed that the P-gp band peaked at 199.8 ± 2.5 kDa; the peak for β-actin was detected at 49.7 ± 2.6 kDa. The bandwidth for P-gp was 24.8 ± 1.9 kDa and ranged from 187.4 kDa to 212.2 kDa; the bandwidth for β-actin was 9.3 ± 1.2 kDa and ranged from 45.1 kDa to 54.35 kDa. In these electropherograms, each AUC is proportional to the amount of protein, allowing comparisons of P-gp levels between CNI and AD patients. Utilizing this method, we found that P-gp protein levels in capillaries isolated from brain tissue of AD patients are 37% lower compared to P-gp protein levels in capillaries isolated from brain samples of CNI (AUC P-gp AD 349670 vs. AUC Pgp CNI 557147 photons × kDa/s). Figure 1E shows an overlay of the electropherograms in Figure 1D. This overlay shows the difference in peak heights and the areas under the curve (AUC) that represent total protein amount for P-gp and β-actin, respectively, which is a more accurate approach to quantitate protein amount than optical density measurements (O’Neill et al., 2006).

To determine ubiquitinated P-gp levels, we performed immunoprecipitation experiments with brain capillaries from brain tissue of CNI and AD patients and observed that ubiquitination of P-gp was 2.8-fold higher in brain capillaries from patients with AD compared to CNI (*n* = 3; *p* = 0.05, Figure 1F; Supplementary Figures S1A,B). This indicates that blood-brain barrier P-gp is highly ubiquitinated in patients with AD.

### PYR41 Prevents Reduction of P-gp Expression and Activity Levels in hAPP Mice

Ubiquitination of a target protein is carried out by three enzymes. First, ubiquitin is activated by the ubiquitin-activating enzyme E1. In the second step, ubiquitin is transferred onto the target protein by the conjugating enzyme E2. This ubiquitin transfer is completed in a third step by the ubiquitin ligase E3 resulting in ubiquitination of the target protein.

**Figure 2:**
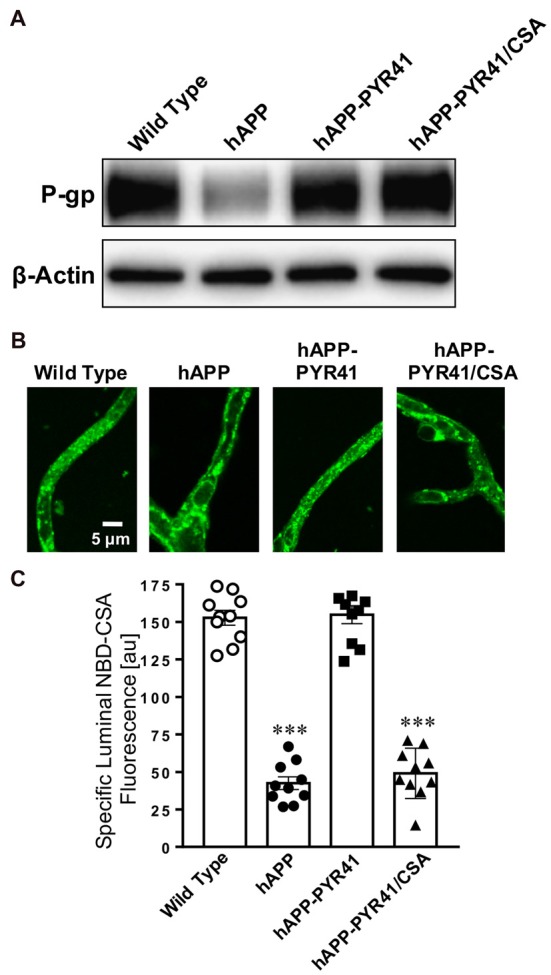
PYR41 prevents reduction of P-gp protein expression and transport activity in human amyloid precursor protein (hAPP) mice. **(A)** Western blot showing that PYR41 treatment restored P-gp protein expression levels in hAPP mice compared to vehicle-treated wild type (WT) mice; β-actin was used as protein loading control. **(B)** Representative confocal images of brain capillaries isolated from WT, hAPP, hAPP-PYR41 and hAPP-PYR41/CSA mice after a 1 h incubation (steady state) with the fluorescent, P-gp-specific substrate NBD-CSA (2 μM). **(C)** Data from ImageJ analysis of confocal images in **(B)** show that luminal NBD-CSA fluorescence was significantly reduced in brain capillaries from hAPP and hAPP-PYR41/CSA mice relative to brain capillaries from WT and hAPP-PYR41 mice. Specific NBD-CSA fluorescence is the difference between total luminal fluorescence and fluorescence in the presence of the P-gp-specific inhibitor PSC833, representing transport activity that is specific to P-gp. Data are mean ± SEM (*n* = 10 capillaries per treatment group); arbitrary fluorescence units (scale 0–255). Statistics: ***p < 0.001 analysis of variance (ANOVA), significantly lower than WT control group.

PYR41 is an E1 ubiquitin-activating enzyme-specific inhibitor that shows little activity on E2 and E3. PYR41 irreversibly blocks ubiquitination, thereby preventing ubiquitin-mediated proteasomal degradation *in vitro* and *in vivo* (Yang et al., 2007; Guan and Ricciardi, 2012). We used PYR41 to test the hypothesis that blocking ubiquitination protects blood-brain barrier P-gp from degradation in transgenic hAPP mice *in vivo*. We dosed young, 8-week old hAPP mice with 2 mg/kg PYR41 i.p. once every 3 days for 14 days (Table 1). An additional group of mice received PYR41 in combination with the P-gp inhibitor cyclosporin A (CSA; 25 mg/kg, p.o.) on days between dosing PYR41 alone. These PYR41/CSA-treated mice serve as control group for P-gp transport activity and are used to account for PYR41-treatment effects that depend on P-gp transport activity. WT and hAPP control mice received vehicle. Treatment of hAPP mice with PYR41 restored P-gp protein expression levels in brain capillary membranes to levels observed in vehicle-treated WT mice (Figure 2A). We also observed this effect in PYR41/CSA-treated animals. Optical density measurements of P-gp (*n* = 3, normalized to β-actin) given as percent of WT control mice (SEM, *p*-value) are: hAPP: 46 ± 10.5% (*t* = 3.52, *p* = 0.022; ANOVA *post hoc* test); hAPP-PYR41: 106 ± 11.8% (*t* = 0.39, *p* = 0.72; ANOVA *post hoc* test), and hAPP-PYR41/CSA: 106 ± 10.4% (*t* = 0.39, *p* = 0.70; ANOVA *post hoc* test).

Next, we determined P-gp transport activity in isolated brain capillaries by using a transport assay we previously described (Hartz et al., 2008, 2010, 2016, 2017). In this assay, freshly isolated brain capillaries are incubated with the fluorescent P-gp substrate NBD-cyclosporin A (NBD-CSA, 2 μM) for 1 h to steady state. Capillaries are then imaged with a confocal microscope followed by quantitative image analysis of NBD-CSA fluorescence in the capillary lumen. In brain capillaries with lower P-gp transport activity compared to control capillaries, less NBD-CSA is transported into the capillary lumen, resulting in lower luminal NBD-CSA fluorescence. Thus, the level of luminal NBD-CSA fluorescence is a measure for P-gp transport activity.

Figure 2B shows representative confocal images of brain capillaries isolated from WT mice, hAPP mice, hAPP mice treated with PYR41 and hAPP mice treated with PYR41/CSA that were exposed to NBD-CSA. Compared to capillaries from WT mice, luminal NBD-CSA fluorescence was decreased in capillaries from hAPP mice and PYR41/CSA-treated hAPP mice, indicating reduced P-gp transport activity levels. In contrast, PYR41 treatment maintained luminal NBD-CSA fluorescence in capillaries from hAPP mice at control levels. Data from confocal image analysis indicate that specific luminal NBD-CSA fluorescence in the lumens of brain capillaries from hAPP mice was reduced by 72% (*t* = 15.1, *p* < 0.0001; ANOVA *post hoc* test) relative to WT mice (Figure 2C). In contrast, luminal NBD-CSA fluorescence levels in brain capillaries from PYR41-treated hAPP mice were similar to levels measured in capillaries from WT mice (*t* = 0.3, *p* = 0.81; ANOVA *post hoc* test). In brain capillaries from PYR41-treated hAPP mice that also received CSA to control for P-gp transport activity luminal NBD-CSA fluorescence was reduced by 68% relative to WT mice; this reduction is comparable to that seen in vehicle-treated hAPP mice (*t* = 13.0, *p* < 0.0001; ANOVA *post hoc* test).

**Figure 3:**
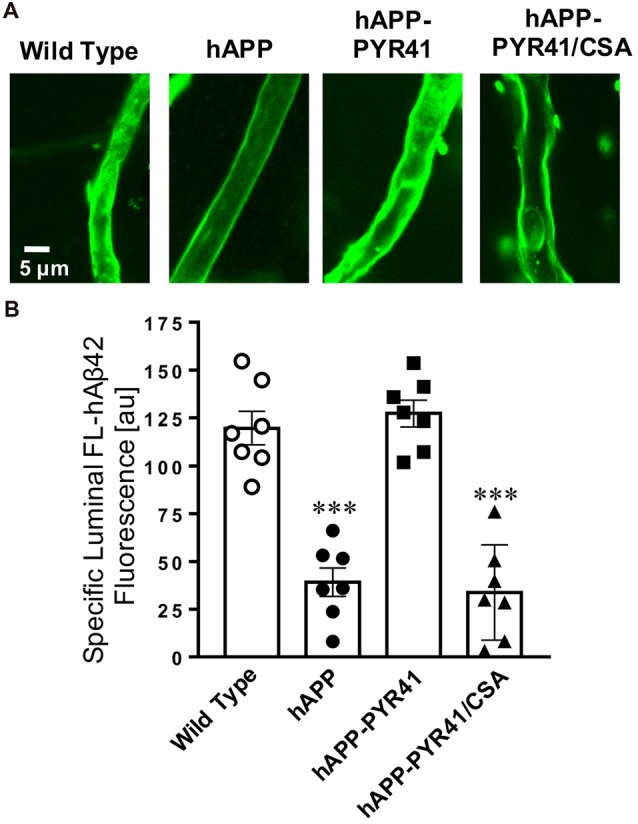
PYR41 treatment restores P-gp-mediated hAβ_42_ transport in hAPP mice. **(A)** Representative confocal images of isolated brain capillaries from WT, hAPP, hAPP-PYR41 and hAPP-PYR41/CSA mice after a 1 h incubation with 5 μM FL-hAβ_42_. **(B)** Data from ImageJ analysis of confocal images in **(A)** show that luminal FL-hAβ_42_ levels were significantly reduced in brain capillaries from hAPP and hAPP-PYR41/CSA mice relative to brain capillaries from WT and hAPP-PYR41 mice. In contrast, luminal FL-hAβ_42_ fluorescence levels in brain capillaries from PYR41-treated hAPP mice are comparable to those in isolated brain capillaries from WT mice indicating that PYR41-treatment restored P-gp transport activity. Specific FL-hAβ_42_ fluorescence is the difference between total luminal fluorescence and fluorescence in the presence of the P-gp-specific inhibitor PSC833, representing FL-hAβ_42_ transport into the lumen of brain capillaries that is specific to P-gp. Data are mean ± SEM (*n* = 7 capillaries per treatment group); arbitrary fluorescence units (scale 0–255). Statistics: ***p < 0.001 (ANOVA), significantly lower than WT control group.

We repeated this experiment using fluorescein-hAβ_42_ (FL-hAβ_42_) to test the ability of P-gp to transport Aβ into the lumen of brain capillaries. Figure 3A shows representative images of isolated brain capillaries incubated to steady state for 1 h with 5 μM FL-hAβ_42_. Image analysis shows that luminal FL-hAβ_42_ fluorescence was reduced to 32.6 ± 9.2% (*t* = 6.4, *p* < 0.0001; ANOVA *post hoc* test) in capillaries from hAPP mice relative to capillaries from WT mice (Figure 3B). Luminal FL-hAβ_42_ fluorescence levels in brain capillaries isolated from PYR41-treated hAPP mice was comparable to fluorescence levels observed in capillaries from WT mice (*t* = 0.6, *p* = 0.54; ANOVA *post hoc* test). In contrast, FL-hAβ_42_ fluorescence levels in capillary lumens from PYR41/CSA-treated control mice were significantly reduced (*t* = 6.87, *p* < 0.0001; ANOVA *post hoc* test), which was comparable to luminal fluorescence levels in brain capillary lumens of untreated hAPP mice.

Together, the data in Figures 2, 3 demonstrate that PYR41 treatment of young hAPP mice attenuated reduction of both P-gp protein expression and transport activity levels. This suggests that PYR41 prevented P-gp degradation through the ubiquitin-proteasome system.

### PYR41 Treatment of hAPP Mice Prevents P-gp Ubiquitination *in Vivo*

**Figure 4:**
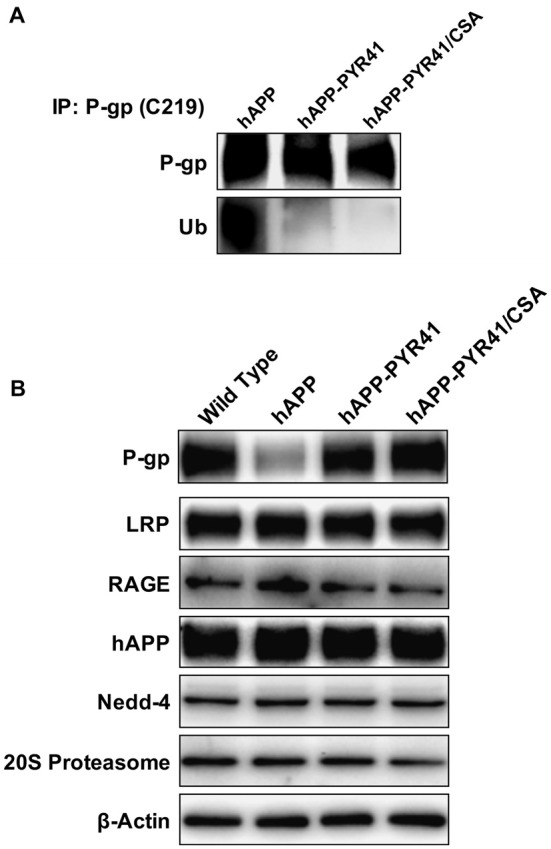
PYR41 prevents P-gp ubiquitination in hAPP mice. **(A)** Western blot showing that treating hAPP mice with PYR41 reduces ubiquitin levels in P-gp-immunoprecipitates from isolated brain capillaries. P-gp was immunoprecipitated in capillary samples isolated from hAPP, hAPP-PYR41 and hAPP-PYR41/CSA mice, and examined for P-gp and ubiquitin levels by Western blotting (brain capillaries isolated from pooled tissue; *n* = 15 mice per treatment group). **(B)** Western blot showing that PYR41 and PYR41/CSA treatment of mice had only an effect on P-gp protein expression levels, whereas levels of LRP, RAGE, hAPP, Nedd-4 and the 20S proteasome were not affected in isolated brain capillaries (pooled tissue; *n* = 15 mice per treatment group).

PYR41 is a cell-permeable, specific and irreversible inhibitor of the ubiquitin-activating enzyme E1, which is responsible for mediating the first step of ubiquitination of proteins that are proteasome targets. Thus, PYR41 inhibits ubiquitination of proteins, thereby preventing their degradation by the ubiquitin-proteasome system. To determine the effect of PYR41 treatment on the ubiquitination status of P-gp at the blood-brain barrier, we performed immunoprecipitation experiments with isolated brain capillaries from PYR41-treated and untreated hAPP mice. The Western blot in Figure 4A shows that immunoprecipitated P-gp protein levels were similar in brain capillaries from hAPP mice and hAPP mice treated with PYR41 and PYR41/CSA. We observed high levels of ubiquitinated P-gp in isolated capillaries from hAPP mice. In contrast, brain capillaries isolated from PYR-41 and PYR41/CSA-treated hAPP mice had no detectable levels of ubiquitinated P-gp. These data indicate that PYR41 treatment was effective to prevent ubiquitination of P-gp in brain capillaries.

The Western blot in Figure 4B shows protein expression levels of P-gp and other proteins involved in Aβ production or transport, as well as signaling molecules associated with P-gp reduction. Data from isolated brain capillaries from hAPP mice showed a reduction in P-gp protein expression levels relative to P-gp levels in WT mice, an effect that was blocked with PYR41 and PYR41/CSA treatment. Importantly, PYR41 and PYR41/CSA treatment did not alter protein expression levels of other proteins associated with Aβ production (hAPP), Aβ clearance (LRP1), Aβ transport (RAGE), or the degradation of P-gp (Nedd-4, 20S Proteasome).

Together, these data demonstrate that PYR41 treatment prevented P-gp ubiquitination in capillaries isolated from hAPP mice but did not affect other proteins involved in Aβ production or transport.

### PYR41 Treatment of hAPP Mice Lowers Aβ Levels

**Figure 5:**
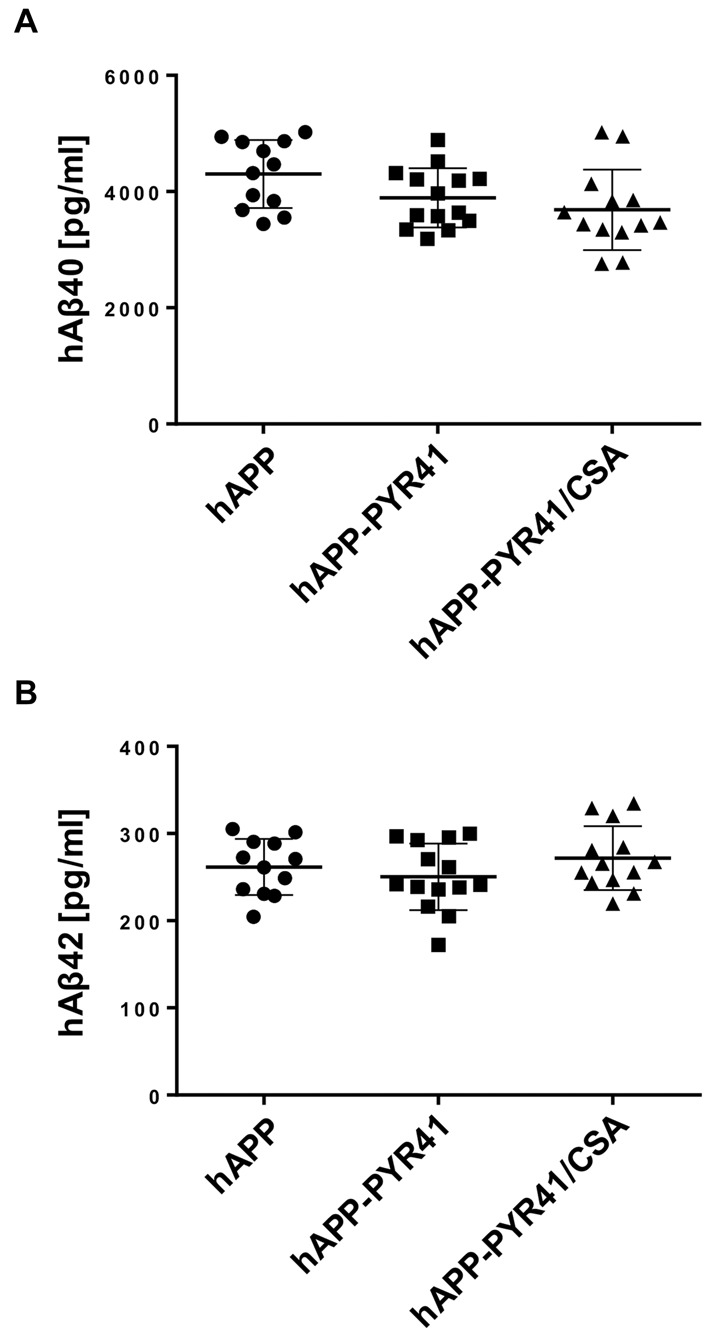
PYR41 treatment has no effect on Aβ plasma levels. **(A)** hAβ_40_ levels (pg/ml) in plasma samples from hAPP mice treated with vehicle, PYR41, or PYR41/CSA determined by ELISA. Data are given for each animal (hAPP: *n* = 12; hAPP-PYR41: *n* = 14; hAPP-PYR41/CSA: *n* = 13). **(B)** hAβ_42_ plasma levels (pg/ml) in samples from hAPP mice treated with vehicle (*n* = 12), PYR41 (*n* = 14), or PYR41/CSA (*n* = 13). Statistics: ANOVA; Data between groups are not significantly different.

In a next step, we determined the consequences of PYR41 treatment on Aβ levels in plasma, capillary membranes, and brain tissue. We measured hAβ_40_ and hAβ_42_ levels in plasma by ELISA and found no difference in hAβ_40_ and hAβ_42_ plasma levels among PYR41-treated, PYR41/CSA-treated, and untreated hAPP mice (Figures 5A,B). Samples from WT mice were not included since WT mice do not express human Aβ.

**Figure 6:**
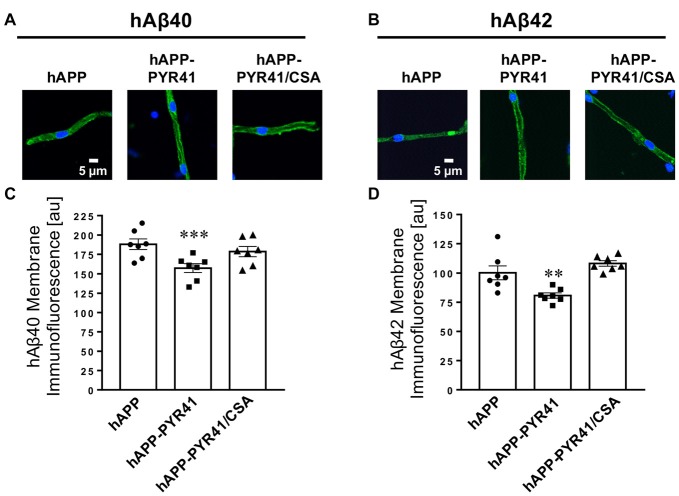
PYR41 treatment reduces Aβ levels in brain capillary membranes. Representative confocal images of **(A)** hAβ_40_-immunostained and **(B)** hAβ_42_-immunostained brain capillaries isolated from vehicle-, PYR41- and PYR41/CSA-treated hAPP mice. Data from image analysis with ImageJ show that **(C)** hAβ_40_ and **(D)** hAβ_42_ membrane immunofluorescence is lower in capillaries isolated from PYR41-treated hAPP compared to control hAPP mice and mice that received PYR41/CSA. Data are mean ± SEM (*n* = 7 brain capillaries per treatment group); shown are arbitrary fluorescence units (scale 0–255). Statistics: **p < 0.01 (ANOVA), ***p < 0.001 (ANOVA) significantly lower than hAPP vehicle-treated mice.

We also immunostained isolated brain capillaries for hAβ_40_ and hAβ_42_ to determine capillary-associated Aβ levels. Brain capillaries isolated from vehicle-treated hAPP mice stained positive for both Aβ peptides (Figures 6A,B). PYR41-treatment decreased membrane-associated immunofluorescence of hAβ_40_ by 16 ± 5.2% (*t* = 8.9, *p* < 0.0001; ANOVA *post hoc* test) and that of hAβ_42_ by 20 ± 2.2% (*t* = 3.1, *p* = 0.0094; ANOVA *post hoc* test) relative to untreated hAPP mice. However, this effect was not observed in Aβ-immunostained brain capillaries from PYR41/CSA-treated hAPP mice, indicating that inhibiting P-gp transport activity with CSA blocks the reduction in Aβ levels in brain capillaries.

**Figure 7:**
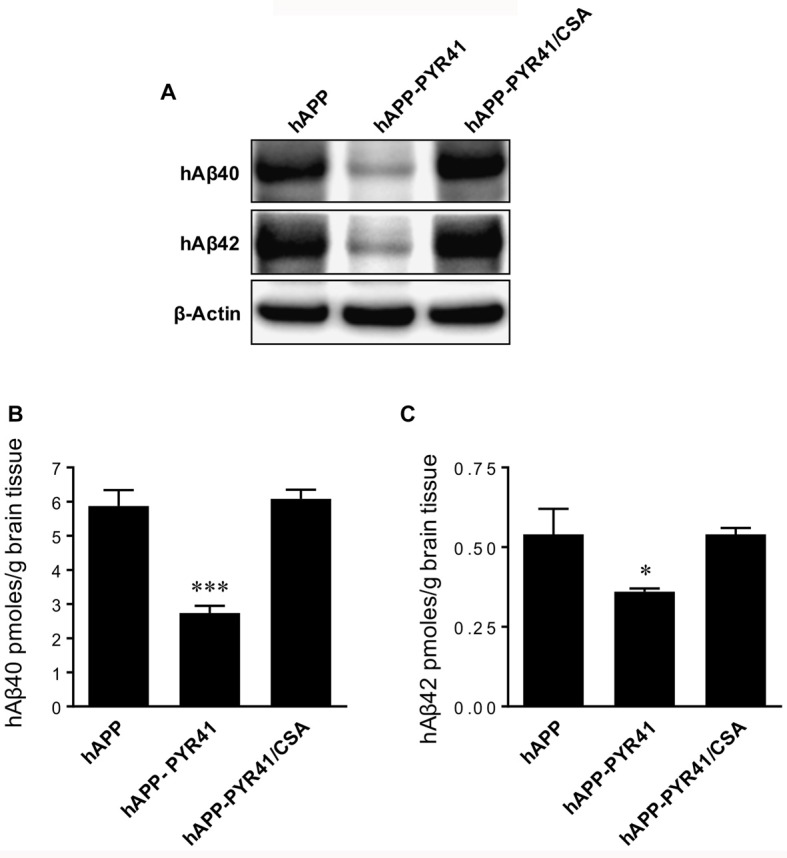
PYR41 treatment reduces Aβ brain levels in hAPP mice. **(A)** Western blots showing PYR41 treatment reduced hAβ_40_ and hAβ_42_ protein expression levels in total brain tissue in hAPP mice compared to vehicle-treated WT mice or hAPP mice treated with PYR41/CSA; β-actin was used as protein loading control. ELISA analysis of brain tissue revealed that PYR41 significantly reduced protein levels of **(B)** hAβ_40_ by 53.3 ± 0.51% and **(C)** hAβ_42_ by 33.3 ± 0.08%. Data are mean ± SEM (pooled tissue from 15 mice per treatment group). Statistics: *p < 0.05 and ***p < 0.001 (ANOVA), significantly lower than hAPP vehicle-treated mice.

Finally, data from Western blot analyses showed a significant reduction of hAβ_40_ and hAβ_42_ protein levels in brain tissue samples of PYR41-treated hAPP mice relative to untreated hAPP mice (Figure 7A). This was not the case in brain tissue from PYR41-treated mice that also received CSA to control for P-gp transport activity. Optical density measurements of Western blots revealed that PYR41 treatment reduced brain hAβ_40_ levels by 42 ± 6.8% (*t* = 6.3, *p* < 0.003; df = 4, *t*-test) and hAβ_42_ levels by 47 ± 4.5% (*t* = 10.5, *p* < 0.0004; df = 4, *t*-test) compared to vehicle-treated hAPP mice. Consistent with our Western blot results, data from ELISA analysis of brain samples also showed a reduction in hAβ_40_ and hAβ_42_ brain levels in hAPP mice treated with PYR41. hAβ_40_ levels were reduced by 53.3 ± 0.51% (*t* = 11.4, *p* = 0.0005; ANOVA *post hoc* test) and hAβ_42_ levels were reduced by 33.3 ± 0.08% (*t* = 4.9, *p* = 0.018; ANOVA *post hoc* test), respectively (Figures 7B,C). Together these data indicate that PYR41 treatment prevents P-gp degradation, which results in a reduction in hAβ_40_ and hAβ_42_ brain levels in hAPP mice.

In summary, our data indicate that blocking P-gp ubiquitination prevents P-gp degradation, which ultimately leads to a reduction in Aβ brain levels. Thus, targeting the ubiquitin-proteasome system early in AD could be a therapeutic strategy to protect brain capillary P-gp and thereby lower Aβ brain levels.

## Discussion

We recently reported that P-gp protein expression and transport activity levels are significantly reduced at the blood-brain barrier in young, 12-week old hAPP mice that do not display cognitive deficits yet (Hartz et al., 2010). We also demonstrated that Aβ_40_ triggers reduction of P-gp expression and activity levels by activating the ubiquitin-proteasome system which leads to degradation of the transporter (Akkaya et al., 2015; Hartz et al., 2016). One consequence of reduced P-gp levels is impaired Aβ clearance from brain to blood across the blood-brain barrier (Hartz et al., 2010). The present study extends our previous *ex vivo* findings to an *in vivo* therapeutic strategy designed to prevent loss of P-gp by targeting the ubiquitin-proteasome system.

Here, we report that P-gp protein expression levels are reduced and P-gp ubiquitination levels are increased in isolated capillaries from AD patients relative to CNIs (Figure 1). Further, we show that treatment of 8–12-week old hAPP mice with PYR41, a specific and irreversible inhibitor of ubiquitin-activating enzyme E1, prevented degradation of P-gp in brain capillaries from hAPP mice (Figure 2). P-gp transport activity levels in PYR41-treated hAPP mice were comparable to those in control WT mice, and P-gp-mediated Aβ_40_ transport activity was also fully restored to control levels in brain capillaries isolated from PYR41-treated hAPP mice (Figure 3). We found that PYR41 treatment reduced levels of ubiquitinated P-gp in brain capillaries from hAPP mice, indicating successful inhibition of E1 function *in vivo* (Figure 4A). Other proteins involved in Aβ production and/or transport were not affected by PYR41 treatment (Figure 4B). While our data suggest that PYR41 treatment prevented P-gp ubiquitination in brain capillaries isolated from hAPP mice without affecting proteins involved in Aβ production or transport (Figure 4B), we cannot fully exclude that PYR41 had no effect on other proteins that could also be involved in Aβ brain accumulation. PYR41 treatment did not affect Aβ plasma levels in hAPP mice, but slightly reduced hAβ_40_ and hAβ_42_ levels in brain capillaries (Figures 5, 6). PYR41 treatment did, however, significantly reduce hAβ brain levels in PYR41-treated hAPP mice compared to vehicle- and PYR41/CSA-treated hAPP control mice (Figure 7).

Together, we provide *in vivo* evidence that inhibiting the ubiquitin-proteasome system blocks the reduction of P-gp levels and lowers Aβ brain levels in an AD mouse model. In the following sections we discuss different aspects of our study and put our findings in context with existing reports.

### The Ubiquitin-Proteasome System Is Involved in P-gp Regulation

The ubiquitin-proteasome system is responsible for degradation of proteins, and therefore, essential for proteostasis (Ciechanover, 1994; Ciechanover and Kwon, 2015). The first step in protein degradation by the ubiquitin-proteasome system is ubiquitination of the target protein that is to be degraded. In a four-step process, a cascade of ubiquitin-activating, -conjugating, -ligating and -elongating enzymes (E1–E4) mediate the conjugation of ubiquitin to an amino group of the target protein. After addition of a polyubiquitin chain on the target protein, the now ubiquitinated membrane protein is internalized, recognized by the 26S proteasome complex and is then degraded in an ATP-driven process. The way proteins are ubiquitinated is complex and multifaceted. Some proteins are tagged with one ubiquitin molecule in a process referred to as monoubiquitination. Other proteins undergo multi-monoubiquitination during which different amino acid residues of the same target protein receive each one ubiquitin molecule. In contrast, polyubiquitination describes a process during which several ubiquitin molecules are added to one target protein resulting in linear or branched polyubiquitinated chains with different topologies. Mono- and polyubiquitination affects proteins in many ways: it can affect protein activity, promote or prevent protein interactions, alter protein cellular location or signal protein degradation via the proteasome.

Our previous work and the present study show that blood-brain barrier P-gp protein expression levels, and thus, P-gp transport activity levels, are regulated by the ubiquitin-proteasome system (Akkaya et al., 2015; Hartz et al., 2016). Our findings suggest that increased proteasomal degradation of P-gp is responsible for reduced P-gp expression and activity levels in AD. Our work is consistent with findings from other groups showing that the ubiquitin-proteasome system regulates localization, protein expression, and transport function of human P-gp (Loo and Clarke, 1998; Zhang et al., 2004). Katayama et al. (2013) demonstrated in the human colorectal cancer cell lines HCT-15 and SW620 that FBXO15, a subunit of the ubiquitin E3 ligase, is a negative regulator of P-gp protein expression. Data from immunoprecipitation experiments indicated that FBXO15 binds to P-gp and enhances ubiquitination of the transporter. FBXO15 knockdown led to increased P-gp expression and transport activity in both cancer cell lines. In the same study, Katayama et al. (2013) also demonstrated that the ubiquitin E3 ligase complex SCFFbx15 recognizes P-gp as a substrate and brings the transporter in contact with the ubiquitin-conjugating enzyme Ube2r1, which ubiquitinates P-gp. More recently, Katayama et al. (2016) showed that inactivating MAPK signaling with small-molecule inhibitors leads to increased Ube2r1 expression levels, which, in turn, promotes P-gp degradation through the proteasome. Ravindranath et al. (2015) showed that the ubiquitin E3-ligase FBXO21 also catalyzes P-gp ubiquitination, thereby targeting it for subsequent proteasomal degradation. In addition, Rao et al. (2006) showed that the E3 ubiquitin ligase, RING finger protein 2 (RNF2), interacts with the linker region of human P-gp and the authors demonstrated that co-expression of RNF2 and P-gp results in decreased ATPase activity and proteolytic protection of the transporter in Sf9 insect cells. Together, data from various groups suggest that P-gp expression and transport activity are regulated by the ubiquitin-proteasome system.

Our previously published work indicates that Aβ_40_ triggers P-gp ubiquitination, resulting in internalization and proteasomal degradation of the transporter (Hartz et al., 2016). Further, we have demonstrated that P-gp may be a substrate for the ubiquitin E3-ligase Nedd-4 (Akkaya et al., 2015). In the present study, we expand this line of research by inhibiting P-gp ubiquitination with the ubiquitin-activating enzyme E1 inhibitor PYR41 in a mouse AD model *in vivo* and show that blocking P-gp ubiquitination prevents loss of P-gp and lowers Aβ brain levels.

In addition to P-gp, other proteins are also thought to facilitate Aβ transport across the blood-brain barrier such as the low-density lipoprotein receptor-related protein (LRP; Deane et al., 2008; Storck et al., 2016). Similar to P-gp, LRP levels were reduced in brain capillaries from various AD mouse models and in post-mortem brain tissue from AD patients (Donahue et al., 2006; Silverberg et al., 2010). Like P-gp, degradation of LRP is mediated by the proteasome, however, LRP degradation appears to be independent from ubiquitination. In this regard, Melman et al. (2002) have demonstrated that tyrosine and di-leucine motifs within the LRP cytoplasmic tail mediate rapid endocytosis followed by proteasomal degradation, a process that does not require ubiquitination. Deane et al. (2004) showed that Aβ enhanced LRP proteasomal degradation in brain capillaries of 6–9-month old hAPP mice with cognitive impairment. The low LRP levels and the behavior deficits in these mice are consistent with reduced LRP levels in Aβ-accumulating mice and patients with AD and familial cerebrovascular β-amyloidosis (Deane et al., 2004). These findings suggest that reduced expression and activity levels of P-gp and LRP in brain capillaries, which contributes to Aβ pathology, result from similar augmentations of the ubiquitin-proteasome system, where P-gp is affected by enhanced ubiquitination and LRP is affected by enhanced direct proteasomal degradation.

### The Ubiquitin-Proteasome System in Alzheimer’s Disease

Protein misfolding, protein mishandling and deficits in protein quality control often drive neurodegenerative disease pathology (Urushitani et al., 2002; Kabashi et al., 2004; Ross and Pickart, 2004). These abnormal processes can lead to accumulation of Aβ in the brain and intraneuronal aggregation of hyper-phosphorylated tau protein, both of which are hallmarks of AD (Oddo, 2008; Riederer et al., 2011; Morawe et al., 2012; Hong et al., 2014; Gentier and van Leeuwen, 2015; Gadhave et al., 2016). Numerous studies reported that the activity of the ubiquitin-proteasome system is reduced in AD. For example, data from post-mortem studies of patients with late-stage AD showed accumulation of ubiquitin in both plaques and tangles and increased levels of a variety of ubiquinated proteins (Perry et al., 1987; Keck et al., 2003). One explanation for this finding is a dysfunctional ubiquitin-proteasome system, where ubiquitinated proteins accumulate in the tissue but are not further degraded by the proteasome. Indeed, Keller et al. (2000) observed a significant decrease in proteasome activity in the hippocampus, parahippocampal gyrus, middle temporal gyri, and inferior parietal lobule of patients with AD compared to cognitive normal individuals. Moreover, in AD, the proteolytic activity of the 26S proteasome appears to be impaired, resulting in oxidation and downregulation of ubiquitin C-terminal hydrolase 1 (UCH1), which is responsible for ubiquitin turnover (Almeida et al., 2006). Further, mutant ubiquitin, UBB^+1^, is a hallmark of various neurodegenerative diseases. Elevated UBB^+1^ levels in the brain directly inhibit proteasome activity, which is thought to lead to Aβ accumulation and hyper-phosphorylated tau, and thus, constitutes a risk factor for AD (Lindsten et al., 2002; van Leeuwen et al., 2006; Shabek et al., 2009; Dennissen et al., 2010; Ciechanover and Kwon, 2015). Collectively, growing evidence suggests that a dysfunctional ubiquitin-proteasome system contributes to AD pathology. However, little is known about the mechanisms that drive irregular activity of the complex ubiquitin-proteasome system, what brain regions and brain cell types are affected is not fully understood, and during which disease stages these changes occur is unclear. In contrast to brain tissue, our previous reports and the present study indicate that in the brain capillaries of the blood-brain barrier, the ubiquitin-proteasome system is overly active, leading to ubiquitination and degradation of membrane proteins such as P-gp (Akkaya et al., 2015; Hartz et al., 2016). The discrepancy between impaired proteasome activity in brain tissue and increased proteasome activity in brain capillary endothelial cells could be due to tissue-specific differences of the 20S proteasome. This explanation is supported by multiple reports showing that enzymes of the ubiquitin pathway (E1–4) are expressed in a tissue-specific manner and that alterations in ubiquitin ligase activity, proteasome subunit composition, and proteasome-interacting proteins are tissue-specific and adapt to functional needs in their respective tissues (Patel and Majetschak, 2007; Mayor and Peng, 2012; Kniepert and Groettrup, 2014; Ortega and Lucas, 2014).

In addition to tissue-specific differences, it is possible that proteasome activity changes with age, and thus, could be time- or even context-dependent. Current studies in our laboratory are aimed at determining levels of ubiquitination and 20S proteasome activity in hAPP mice at different ages.

### The Ubiquitin-Proteasome System as a Therapeutic Target

Collectively, our previous and present findings suggest that the ubiquitin-proteasome system is involved in reducing P-gp protein expression and transport activity levels in AD. Based on data from our studies, preventing P-gp loss by targeting the ubiquitin-proteasome pathway could potentially serve as therapeutic strategy to protect P-gp from degradation, reduce Aβ brain accumulation, and slow AD progression. While targeting ubiquitination may represent a new therapeutic strategy, developing drugs that target the ubiquitin activating, conjugating, ligating and elongating enzymes E1–E4 remains a challenge. These enzymes do not have a well-defined catalytic pocket, which makes the design of specific small molecule inhibitors difficult (Nalepa et al., 2006). Further, ubiquitination is a complex process that depends on dynamic protein-protein interactions that are difficult to disrupt with small molecules. In addition, as the name indicates, ubiquitination is a ubiquitous process present in all cells of the body, and thus, selectively targeting one tissue and not others is challenging. Consequently, FDA-approved drugs that specifically and selectively block ubiquitination are currently not available.

A different therapeutic option could be to target the proteasome itself. Currently, two FDA-approved small-molecule 20S proteasome inhibitors are on the market for cancer therapy: bortezomib (Velcade^®^) and carfilxomib (Krypolis^®^). Both drugs show efficacy in multiple myeloma with manageable pharmacokinetic properties and a relatively low side effect profile. Since the 20S proteasome subunit is the proteolytic core of the multi-subunit 26S proteasome proteolytic complex, both inhibitors—bortezomib and carfilxomib—also affect the activity of the entire 26S proteasome complex. While targeting the proteasome is an option, two points need more reflection. First, the potential inverse relationship between dysfunction of the ubiquitin-proteasome system in the brain relative to the blood-brain barrier must be taken into consideration. Inhibiting the 20S proteasome may be particularly beneficial in the early stages of AD to prevent P-gp loss and facilitate Aβ clearance. However, 20S proteasome inhibition may be detrimental in later AD stages when the ubiquitin-proteasome system is greatly impaired in the brain. Second, 20S proteasome inhibition is only feasible and can only be fully taken advantage of when AD is diagnosed early. While significant efforts have been made to identify biomarkers and develop novel imaging techniques that allow early AD diagnosis, no tools are currently available in the clinic and early AD diagnosis, prior to the appearance of behavioral symptoms, remains a formidable challenge.

In summary, in the present *in vivo* study we show that preventing ubiquitination of P-gp at the blood-brain barrier protects the transporter from degradation, which substantially lowers Aβ brain levels in an AD mouse model. These data suggest a novel therapeutic avenue that helps protect P-gp by limiting Aβ-induced P-gp degradation for improved Aβ clearance across the blood-brain barrier in AD.

## Author Contributions

AH and BB contributed to the major design, acquisition, analysis and interpretation of data for the work and wrote and revised the manuscript. YZ carried out ELISAs and experiments with isolated human capillaries. AS contributed to Western blot data analysis and drafted parts of the manuscript. EA provided all statistical analyses. All authors were involved in drafting and revising the work for important intellectual content. All authors approved the final version and agreed to be accountable for all aspects of the work in ensuring that questions related to the accuracy or integrity of any part of the work are appropriately investigated and resolved.

## Conflict of Interest Statement

The authors declare that the research was conducted in the absence of any commercial or financial relationships that could be construed as a potential conflict of interest.
