# Supplementary material for: Preventing P-gp Ubiquitination Lowers Aβ Brain Levels in an Alzheimer’s Disease Mouse Model
Source: Front Aging Neurosci. 2018 Jun 26;10:186. doi: 10.3389/fnagi.2018.00186 (PMC6028735; doi:10.3389/fnagi.2018.00186)
Supplement: Supplementary file 1 [file Data_Sheet_1.PDF]

Supplemental Figure 1

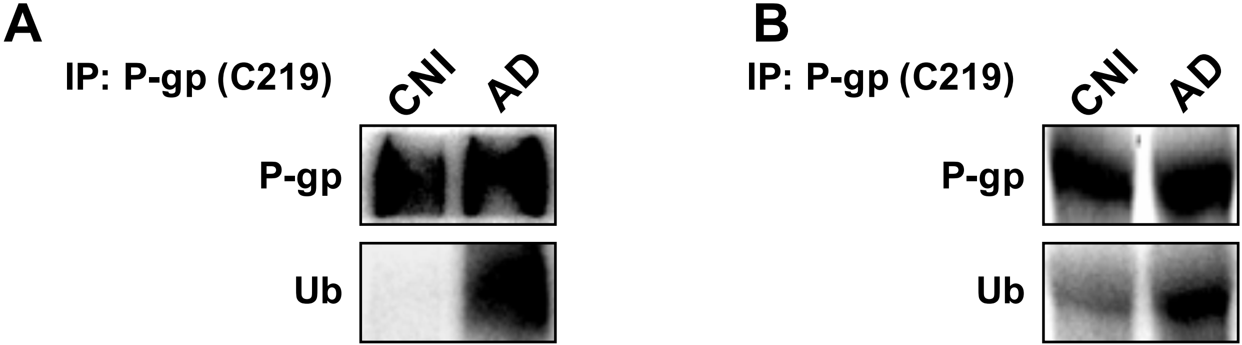

Supplemental Figure 1 . P-gp ubiquitination levels are increased in brain capillaries from AD patients. A, B) Western blot showing that ubiquitin levels in P-gp-immunoprecipitates are increased in capillaries from AD patients compared to those from CNI. Capillaries were isolated from human brain tissue (frontal cortex) of AD patients versus cognitive normal individuals.
